# Protein Profiling of Arabidopsis Roots Treated With Humic Substances: Insights Into the Metabolic and Interactome Networks

**DOI:** 10.3389/fpls.2018.01812

**Published:** 2018-12-12

**Authors:** Sohaib Roomi, Antonio Masi, Giovanni Battista Conselvan, Sara Trevisan, Silvia Quaggiotti, Micaela Pivato, Giorgio Arrigoni, Tayyaba Yasmin, Paolo Carletti

**Affiliations:** ^1^Department of Biosciences, COMSATS University Islamabad, Islamabad, Pakistan; ^2^Department of Agronomy, Food, Natural Resources, Animals and Environment, University of Padua, Padua, Italy; ^3^Proteomics Center, University of Padua and Azienda Ospedaliera di Padova, Padua, Italy; ^4^Department of Biomedical Sciences, University of Padua, Padua, Italy

**Keywords:** proteomics, biostimulant, iTRAQ, ubiquitin, cell wall, redox homeostasis

## Abstract

**Background and Aim:** Humic substances (HSs) influence the chemical and physical properties of the soil, and are also known to affect plant physiology and nutrient uptake. This study aimed to elucidate plant metabolic pathways and physiological processes influenced by HS activity.

**Methods:** Arabidopsis roots were treated with HS for 8 h. Quantitative mass spectrometry-based proteomics analysis of root proteins was performed using the iTRAQ (Isobaric Tag for Relative and Absolute Quantification) technique. Out of 902 protein families identified and quantified for HS treated vs. untreated roots, 92 proteins had different relative content. Bioinformatic tools such as STRING, KEGG, IIS and Cytoscape were used to interpret the biological function, pathway analysis and visualization of network amongst the identified proteins.

**Results:** From this analysis it was possible to evaluate that all of the identified proteins were functionally classified into several categories, mainly redox homeostasis, response to inorganic substances, energy metabolism, protein synthesis, cell trafficking, and division.

**Conclusion:** In the present study an overview of the metabolic pathways most modified by HS biological activity is provided. Activation of enzymes of the glycolytic pathway and up regulation of ribosomal protein indicated a stimulation in energy metabolism and protein synthesis. Regulation of the enzymes involved in redox homeostasis suggest a pivotal role of reactive oxygen species in the signaling and modulation of HS-induced responses.

## Introduction

Humic substances (HSs) are complex, heterogeneous, and widely distributed mixtures of organic compounds with different functional groups and molecular masses. These compounds represent the end products of microbial decomposition and chemical degradation of dead biota and are considered the major components of soil organic matter (Nardi et al., 2002) and the most abundant naturally occurring organic molecules on Earth (Sutton and Sposito, 2005). Due to the structural complexity they are yet to be separated into pure components (Muscolo et al., 2013) while they are traditionally obtained from soils or sediments by means of dilute base solutions (Schnitzer and Monreal, 2011).

Humic substance influence the chemical and physical properties of the soil and its overall health as they participate in many agronomic, environmental, and geochemical processes (Nardi et al., 2009). HS can be used directly on plants at low concentrations (Aguiar et al., 2012) to enhance plant growth, yield and nutrient uptake. For this reason, HS constitute a category of plant biostimulants as defined in du Jardin (2012) and Calvo et al. (2014): studying plant responses to these compounds might have a broader impact, elucidating the effects of biostimulants in general.

The mechanisms of action of HS and other biostimulants are still debated. HS have been found to affect plant growth in different ways such as interacting with morphological and physiological processes related to plant growth (Vaughan and Malcom, 1985; Yang et al., 2004; Jindo et al., 2011; Conselvan et al., 2017), lateral root development and root hair formation (Nardi et al., 2009), and root cell elongation (Canellas and Olivares, 2014). HS are also reported to upregulate glycolysis and tricarboxylic acid cycle (TCA) (Conselvan et al., 2018) and nitrate uptake and metabolism (Quaggiotti et al., 2004).

Some authors demonstrated that these positive effects could be due to an auxin-like effect (Trevisan et al., 2010; Canellas et al., 2011; Mora et al., 2014), while Nitric oxide signaling (Zandonadi et al., 2010) has been proposed to be involved in HS-induced increase in plasma membrane (PM) H^+^-ATPase activity in the root (Canellas et al., 2002). In parallel with these studies other research presented metabolic changes of reactive oxygen species (ROS) in plants after root application of humic acids (HAs) from vermicompost (García et al., 2012, 2014). Although ROS are mainly recognized as toxic chemical species deriving from aerobic metabolism (Mittler et al., 2011), they have been designated as signaling molecules involved in transduction mechanisms controlling metabolic processes like plant growth and development (Foreman et al., 2003; Berbara and Garciìa, 2014; Mittler, 2017). In this context ROS have been proposed as mediators of the effects of HS in plants (Garcia et al., 2016).

In the last decade, new molecular “omics” approaches have been used to characterize the complexity of signal cascades and biochemical reactions responsible for the beneficial effects of HS on plant metabolism. Just a few papers (Carletti et al., 2008; Trevisan et al., 2011; Jannin et al., 2012; Gao et al., 2015; Conselvan et al., 2018) have been published using these techniques for analyzing plant responses to HS. From the fundamental research perspective, studies on the transcriptomics and proteomics effects of HS will help to clarify how these biostimulants elicit plant growth, nutrient uptake, and stress-tolerance responses. Zandonadi et al. (2013) recently evidenced the need of efforts directed toward the development of standardized, accessible and cost-effective methods to quantify and qualify the biostimulant bioactivity. Such studies also would offer the potential to find biological markers to be used during product development (Calvo et al., 2014).

The aim of the present work was to understand the physiological mechanisms underlying plant responses to HS. Plant growth parameters and protein content were recorded to assess Arabidopsis response to HS treatment. iTRAQ (Isobaric Tag for Relative and Absolute Quantification) technique coupled to liquid chromatography-mass spectrometry (LC–MS) analysis was applied to evaluate the changes in the proteome of Arabidopsis roots following HS treatment. Present results have been compared with recent transcriptomic and metabolomic data obtained in analogous experimental conditions. Finally, quantitative reverse transcription PCR (qRT-PCR) has been performed to assess the transcript accumulation of three genes encoding differentially abundant proteins evidenced by iTRAQ.

## Materials and Methods

### Preparation of Humic Extract

The *feces* of *Nicodrilus* [ = *Allolobophora* (Eisen) = *Aporrectodea* (Oerley)] *caliginosus* (Savigny) and *Allolobophora rosea* (Savigny) (Minelli et al., 1995) were collected from the Ah horizon of an uncultivated couchgrass, *Agropyron repens* L., growing in soils classified as Calcaric Cambisol (CMc-F.A.O. classification) (FAO-UNESCO, 1997). Earthworm culture conditions, HS extraction, and extract purification were conducted as reported in Carletti et al. (2008). HS extraction was performed with 0.1N KOH. The extract was desalted by using 14 kDa cut-off dialysis Visking (Medicell, London, United Kingdom) tubing against distilled water. Subsequently, the extract was desalted on ion exchange Amberlite IR-120 (H^+^ form), assessed for organic carbon content, and lyophilized before conducting the following analyses. Humic substances chemical characterization can be retrieved in Conselvan et al. (2018).

### Plants Growth and Treatment Conditions

Arabidopsis (*Arabidopsis thaliana*) plants were hydroponically grown in a growth chamber as described previously (Destro et al., 2011). After vernalization, *A. thaliana* wild-type seeds were germinated in the dark before being transferred to the hydroponic system. Seedlings were grown in pools containing Murashige and Skoog basal salt mixture (Sigma-Aldrich) solution for 28 days, and the medium was changed every 7 days. Growth conditions were: 14 h of light at 20°C, 10 h of dark at 18°C and constant 60% relative humidity. After 28 days of pre-cultivation, plants were partitioned in two hydroponic system batches: one, containing Murashige and Skoog solution, was kept as control (CTRL), in the other 1 mg C/L of HS from earthworm *feces* was added to the solution. After 8 h of treatment, about 200 mg of plant roots (12 plants) were pooled and collected for each treatment, snap frozen in liquid nitrogen, and immediately treated for extraction. Three independent biological replicates were performed. Plant fresh root and leaves weights were recorded during sample collection in triplicate for each biological replicate (*n* = 9). Root images were collected using a flatbed scanner. The primary root length was measured using the Image J Image Analysis Software. Three biological replicates for each treatment and an ANOVA statistic test were performed (*n* = 30). Analysis of variance (one-way ANOVA) was performed using the SPSS 23 (IBM, Corp.) software.

### Protein Content Determination

Fresh leaf and root samples, previously stored at -80°C, were ground to a homogenous powder with liquid N_2_. Proteins were extracted by homogenizing 0.2 g of root or shoot materials with 5 mL of 38 mM KH_2_PO_4_ and 62 mM K_2_HPO_4_, pH 7, at 4°C. After 2 min, the extract was filtered through three layers of muslin and centrifuged at 15,000 *g* for 20 min at 4°C. A 50-μL supernatant sample was incubated with 50 μL of Milli-Q water and 2.5 mL of 0.00117 M Bradford reagent. The protein concentration in the extract was determined according to Bradford (1976), after 15 min of incubation, using a Jasco V-530 UV/Vis spectro- photometer (Jasco Corporation, Tokyo, Japan) at 595 nm wavelength. Three independent biological replicates were analyzed three times for a total of nine replicates. The protein concentration was expressed as mg of protein per g of fresh root or leaf. Analysis of variance (one-way ANOVA) was performed using the SPSS 23 (IBM, Corp.) software.

### RNA Extraction and cDNA Synthesis

RNA was extracted from 12 pooled seedlings, in three independent biological repetitions, and extracted using TRIzol reagent (Invitrogen, Thermo Fisher Scientific, Waltham, MA United States) as previously described by Trevisan et al. (2011). RNA was quantified with a Nanodrop1000 (Thermo Scientific, Nanodrop Products, Wilmington, DE, United States) and reverse transcribed to cDNA as described by Manoli et al. (2012).

### Quantitative Reverse Transcription PCR (qRT-PCR)

Quantitative reverse transcription PCR (qRT-PCR) was performed as described by Nonis et al. (2007) using the StepOne Real-Time PCR System (Applied Biosystems, Thermo Fisher Scientific, Waltham, MA, United States). Melting-curve analysis confirmed the absence of multiple products and primer dimers. The gene-specific primers for AT4G35830, AT1G57720, and AT3G60770 were designed with Primer3 software version 0.4.0^1^ (AT4G35830for: GCGTTAGAGAAGCCTGATGG; AT4G35830rev: CTCAACCTGCTTGGGAGAAG; AT1G57720 for:CACTCTGTCACCCTTGCTGA, AT1G57720rev:GCATCA CCCAACACCTTCTT; AT3G60770for:GCTCAAGACAACC CCTCAAG, AT3G60770rev:TCTCAAGATGCTTGCGGATA). Gene expression values were normalized to the 18S gene and reported as arbitrary units (AUs) of mean normalized expression (Trevisan et al., 2010). Three technical replicates were performed on three independent biological repetitions.

### Root Protein Extraction

Protein extraction was performed as described by Lan et al. (2011). Briefly, roots (ca. 150–250 mg) were ground in liquid nitrogen; 50 mL pre-cooled acetone (-20°C), 10% trichloroacetic acid (TCA) and 0.07% 2-mercaptoethanol were added and mixed vigorously. After 2 h of precipitation at -20°C, proteins were collected by centrifuging at 35,000 *g* (JA-20 rotor; Beckman Coulter Avanti J-E) at 4°C for 30 min. The supernatants were removed, and the protein pellets were washed twice with cold acetone containing 0.07% 2-mercaptoethanol and 1 mM phenylmethanesulfonyl fluoride and a third time with cold acetone without 2-mercaptoethanol. Protein pellets were extracted using protein extraction buffer composed of 6 M urea, 50 mM triethylammonium bicarbonate, pH 8.5, and 2% CHAPS. In addition to the protocol of Lan et al. (2011), 1% PVPP was added to the extraction buffer to improve the purification from impurities such as polyphenols, and four cycles of sonication (10″ at 72 Hz, 10″ pause) were performed to improve protein solubilization. Protein extracts were then centrifuged at 19,000 *g* (JA-20 rotor; Beckman Coulter Avanti J-E) for 20 min at 4°C. Eventually, supernatants were collected, and the protein concentrations were determined using a protein Bradford assay kit (Sigma Aldrich). Extracted proteins were then precipitated overnight with 80% acetone at -20°C.

### *In situ* Trypsin Digestion and iTRAQ Labeling

To further clean the sample from the detergent present in the extraction buffer, protein pellets were loaded into a pre-cast 4–12% SDS gel and the electrophoretic run was stopped as soon as the protein extracts entered the running gel. They were then excised from the gels as single, narrow bands, and *in situ* digestion and peptide extraction were performed according to Resmini et al. (2017). The resulting peptide solution was desalted on a C18 solid-phase extraction cartridge and 1 μg of each sample was analyzed by LC–MS/MS to check the digestion efficiency (details of the instruments and instrumental methods are given in the following section). Peptides belonging to the two studied conditions (control and HS) were labeled with the iTRAQ reagents (ABSciex) according to the manufacturer’s instructions, and for the three biological replicates a tag swapping strategy was used following the Latin square experimental design. Prior to mixing the samples in a 1:1 ratio, 1 μg of each sample was analyzed separately by LC–MS/MS (details of the instruments and instrumental methods are given in the following section). The resulting data were searched against the database, setting the iTRAQ labeling as a variable modification. All peptides were correctly identified as being iTRAQ-modified at the N-terminus and at each lysine residue. The samples were then pooled and dried under vacuum for further analysis.

### Strong Cation Exchange Fractionation

Strong cation exchange chromatography was performed on a strong cation exchange cartridge (AB Sciex) as previously described (Tolin et al., 2013). The labeled samples were dissolved in 500 μl of buffer A (10 mM KH_2_PO_4_, 25% acetonitrile, pH 2.9) and loaded onto the cartridge using a syringe pump with a 50 μL/min flow rate. The cartridge was washed three times with 500 μL of buffer A. Peptides were eluted in a stepwise manner with increasing concentrations of KCl in buffer A. The labeled peptides were eluted in eight fractions (500 μL per fraction) with the following concentrations of KCl in buffer A: 50, 100, 120, 140, 160, 180, 200, and 350 mM. The volume of each fraction was reduced under vacuum to remove acetonitrile. Samples were desalted using C18 cartridges (Sep-Pack, C18, Waters, Milford, MA, United States) according to the manufacturer’s instructions. Samples were finally dried under vacuum and kept at -20°C until MS analysis.

### LC–MS/MS Analysis

Samples were re-suspended in H_2_O/0.1% formic acid and 1 μg of each fraction underwent LC–MS/MS analysis. The MS analyses were conducted with a LTQ-Orbitrap XL mass spectrometer (Thermo Fisher Scientific, Pittsburgh, CA, United States) coupled online with a nano-HPLC Ultimate 3000 (Dionex – Thermo Fisher Scientific). Samples were loaded onto a homemade 10 cm chromatographic column packed into a pico-frit (75 μm id, 10 μm tip, New Objectives) with C18 material (ReproSil, 300 Å, 3 μm). Peptides were eluted with a linear gradient of acetonitrile/0.1% formic acid from 3 to 50% in 90 min at a flow rate of 250 nL/min. According to the method described by Kocher et al. (2009), the instrument performed a full scan at high resolution (60,000) on the Orbitrap, followed by MS/MS scans on the three most intense ions with CID fragmentation on the linear trap. MS/MS scans were performed on the same ions with higher energy collision dissociation fragmentation (HCD) on the Orbitrap (with a resolution of 7,500) to obtain low mass range data suitable for protein quantification. Peptides reliably identified in each sample were inserted in a static exclusion list that was used to perform (under the same chromatographic and instrumental conditions) a second LC–MS/MS run for each sample fraction.

### Database Search and Quantification

Raw LC–MS/MS files were analyzed using Proteome Discoverer 1.4 (Thermo Fisher Scientific). The software was connected to a Mascot Search Engine server, version 2.2.4 (Matrix Science, London, United Kingdom). The spectra were searched against an *A. thaliana* database (downloaded from ARATH UniProt database version dated January 2013) with a MudPIT protocol. Enzyme specificity was set to trypsin with two missed cleavages. Peptide and fragment tolerance was set to 10 ppm and 0.6 Da, respectively. Carbamidomethylation of cysteines, 4-plex iTRAQ at the *N*-terminus and Lys were set as fixed modifications, while methionine oxidation was selected as a variable modification. Based on the search against the corresponding randomized database, false discovery rates (FDRs) were calculated by the software.

The data were pre-filtered to exclude MS/MS spectra containing less than five peaks or with a total ion count below 50. All proteins identified with at least two independent unique peptides and with FDR ≤ 5% were considered as positive hits and grouped into protein families according to the principle of maximum parsimony (all relevant information regarding protein and peptide identification and quantification are reported in Supplementary Tables S2, S3). The mass spectrometry proteomics data have been deposited to the ProteomeXchange Consortium via the PRIDE partner repository (Vizcaíno et al., 2016) with the dataset identifier PXD009989. A two-tailed *Z*-test was used to highlight proteins with a significantly (*p* ≤ 0.05) different abundance in treated vs. control roots. A fold change of treated to control ≥ 1.3 was set as the threshold for increased abundance, while a fold change ≤-1.3 was taken to indicate decreased protein content.

### Bioinformatics Analysis

Identified proteins were analyzed by means of KEGG Mapper – Search&Color Pathway on-line tool^2^ (Kanehisa et al., 2016, 2017) against *A. thaliana* database using UniProt ID as object.

Networks of functionally related proteins were created using STRING version 10.5 (Szklarczyk et al., 2017), while the tool GeneCodis3 (Tabas-Madrid et al., 2012) was used to highlight biological annotations significantly associated to the list of differentially abundant proteins.

Protein interactomes were built with Integrate Interactome System (IIS) platform^3^ (Carazzolle et al. (2014). Interactome network was built based only on differentially expressed proteins, expanding the network to first neighbors’ nodes (Bernardo et al., 2017) (Figure 3). IIS output networks were visualized and analyzed using Cytoscape 3.5.1 software (Shannon et al., 2003).

For functional analysis of differentially regulated transcripts and proteins, targets were classified according to GO terms using the PANTHER (Mi et al., 2013). Classification System Graph-based visualization of GO categories and interactive graphs were developed by REVIGO (Supek et al., 2011).

## Results

**FIGURE 1:**
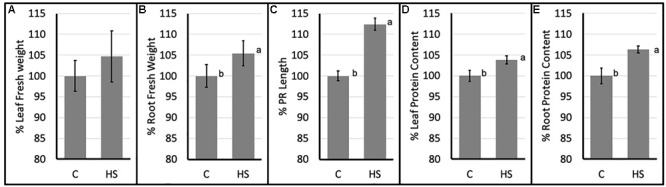
Percent values (mean ± SE) of leaf **(A)** and root **(B)** fresh weights; principal root length **(C)** and leaf **(D)** and root **(E)** protein content of *Arabidopsis thaliana* (C, control; HS, humic substances treated). Data are mean ± SD, *n* = 9. Letters indicate significant differences among treatments (*p* ≤ 0.05) based on one-way ANOVA.

Significant differences in morphological parameters were evidenced in HS treated Arabidopsis plants compared to control. In particular both root fresh weight and principal root length resulted higher following HS treatment. Moreover, total protein content (Figures 1D,E) resulted significantly higher in treated plants roots and aerial parts.

Following iTRAQ labeling, mass spectrometry analysis of HS treated samples of Arabidopsis roots resulted in identification of 902 different protein groups in the three biological replicates. Supplementary Table S1 lists all proteins with their quantification values and all parameters related to MS analysis. Following statistical analysis, proteins were considered as significantly altered (*p*-value ≤ 0.05) with a fold-change ≥ 1.3 (up regulated proteins) or with a fold-change ≤-1.3 (down-regulated proteins). Sixty three identified proteins were found to have an increased abundance (Table 1); while 29 proteins resulted to have lower abundance compared to the control (Table 2).

**Table 1:** List of proteins with increased abundance in HS treated vs. untreated roots.

UniProt ID	Locus name	Description	Fold change	Coverage (%)	Unique peptides	Localization
Q9XI10	AT1G21680	DPP6 N-terminal domain-like protein	2.3	3.3	3	CW, V, P,
Q56ZI2	AT1G22530	Patellin-2	2.3	26.5	12	P, CH,
Q84WU7	AT3G51330	Aspartyl protease family protein	2.3	5.1	3	P
Q9ZPI1	AT3G11710	Lysine–tRNA ligase	2.1	6.6	4	
Q9C8Y9	AT1G66280	Beta-glucosidase 22	2.1	43.7	17	ER, CH,
F4J9K9	AT3G05900	Neurofilament protein-related protein	2.0	34.8	18	C
P51418	AT2G34480	60S ribosomal protein L18a-2	2.0	22.5	6	C, R, P,
F4HV16	AT1G47600	Myrosinase 4	2.0	6.5	3	EM
Q8RX87	AT5G20250	Probable galactinol-sucrose galactosyltransferase 6	2.0	4.1	3	CH
Q9M8T0	AT3G02880	Probable inactive receptor kinase At3g02880	2.0	14.2	8	CW, P,
Q9FJ62	AT5G55480	Probable glycerophosphoryl diester phosphodiesterase 1	2.0	10.4	7	P
Q9SZ51	AT4G31840	Early nodulin-like protein 15	2.0	22.0	5	P
O82762	AT2G25970	F17H15.1/F17H15.1	2.0	18.0	9	C
Q9STW6	AT4G24280	Heat shock 70 kDa protein 6	1.9	23.4	4	M, CH
Q56WK6	AT1G72150	Patellin-1	1.9	24.3	12	EC, V, P, CH,
Q9S791	AT1G70770	AT1G70770 protein	1.9	7.7	4	ER, P
Q9SR37	AT3G09260	Beta-glucosidase 23	1.8	37.2	13	V
Q9M9K1	AT3G08590	Probable 2,3-bisphosphoglycerate-independent phosphoglycerate mutase 2	1.8	12.3	4	EC, C
F4K0F7	AT5G60640	Protein disulfide-isomerase A1	1.8	29.1	13	M, ER, P, CH
O22126	AT2G45470	Fasciclin-like arabinogalactan protein 8	1.8	23.3	9	EC, CW, P, A
Q9C525-2	AT1G66270	Isoform 2 of Beta-glucosidase 21	1.7	37.9	10	V
F4J110	AT3G63460	Protein transport protein SEC31	1.7	4.0	4	C, G, MM
F4JBY2	AT3G60750	Transketolase	1.6	10.8	6	CH
O23006	AT2G17120	LysM domain-containing GPI-anchored protein 2	1.6	19.4	8	P
Q9SRH6	AT3G01290	Hypersensitive-induced response protein 3	1.6	21.1	6	M, V, P
Q39043	AT5G42020	Mediator of RNA polymerase II transcription subunit 37f	1.6	49.6	7	CW, Nu, V, ER, P,
F4JWM1	AT5G18380	40S ribosomal protein S16-3	1.6	41.7	6	C, R, CH,
Q42560	AT4G35830	Aconitate hydratase 1	1.5	19.9	9	EC
Q9FVT2	AT1G57720	Probable elongation factor 1-gamma 2	1.5	14.3	7	CW, V
Q9SMT7	AT3G48990	4-Coumarate-CoA ligase-like 10	1.5	21.0	12	EC, CH
F4IB69	AT1G51850	Leucine-rich repeat protein kinase family protein	1.5	3.8	4	P
Q9FXA2	AT1G49760	Polyadenylate-binding protein 8	1.50	21.9	11	C, N
O50008	AT5G17920	5-Methyltetrahydropteroyltriglutamate-homocysteine methyltransferase	1.5	45.6	20	EC, PO, C, P
Q56ZQ3	AT2G14720	Vacuolar-sorting receptor 4	1.5	17.0	10	V, G, P
Q680P8	AT4G33865	40S ribosomal protein S29	1.5	48.2	3	C, R,
C0Z361	AT5G56500	Chaperonin 60 subunit beta 3	1.4	17.1	3	M, CH
P42731	AT4G34110	Polyadenylate-binding protein 2	1.4	26.4	13	C
Q9FJI5	AT5G40760	Glucose-6-phosphate 1-dehydrogenase, cytoplasmic isoform 2	1.4	20.4	12	C
Q9SRG3	AT1G77510	Protein disulfide isomerase-like 1-2	1.4	32.1	8	ER, P, CH,
P0DH99	AT1G07940	Elongation factor 1-alpha 1	1.4	36.1	17	Nu, M, V, P, CH,
Q1H583	AT1G54000	GDSL esterase/lipase	1.4	50.6	15	V, EC, CW, V, P,
F4KHS2	AT5G59090	Subtilase 4.12	1.4	21.8	14	EC
P22953	AT5G02500	Heat shock cognate 70 kDa protein 1	1.4	45.8	12	EC, CW, Nu, C, R, P, CH,
Q8L7E3	AT4G20110	Vacuolar-sorting receptor 7	1.3	22.7	14	G, P
P59223	AT3G60770	40S ribosomal protein S13-1	1.3	41.1	7	CW, Nu C, R, CH
P53492	AT5G09810	Actin-7	1.3	40.1	5	CW, Nu, M, CS, P,
Q9LTF2	AT5G52650	40S ribosomal protein S10-3	1.3	37.4	6	CW, C,R
Q9LX13	AT5G10160	(3R)-Hydroxymyristoyl-[acyl carrier protein] dehydratase-like protein	1.3	13.7	3	CW, CH,
Q9FKK7	AT5G57655	Xylose isomerase	1.3	11.3	6	V, ER, P
O65719	AT3G09440	Heat shock 70 kDa protein 3	1.3	42.2	10	EC, CW, V, C, R, P, CH,
Q9SE60	AT3G59970	Methylenetetrahydrofolate reductase 1	1.3	12.5	4	C
Q9SVG4-2	AT4G20830	Isoform 2 of Reticuline oxidase-like protein	1.3	13.7	8	EC, CW, M, V, P
Q94C59	AT1G30690	Patellin-4	1.3	7.2	3	C, P,
O80517	AT2G44790	Uclacyanin-2	1.3	30.2	5	P
Q9SIB9	AT2G05710	Aconitate hydratase 2	1.3	17.7	9	CW, M, P, CH
Q94A28	AT4G26970	Aconitate hydratase 3	1.3	17.9	14	M, CH
O48773	AT2G32920	Protein disulfide-isomerase 2-3	1.3	10.2	4	ER
F4JMJ1	AT4G16660	Heat shock 70 kDa protein 17	1.3	4.3	3	ER
Q9SGH6	AT3G01420	Alpha-dioxygenase 1	1.3	15.2	7	EC
Q9LZ66	AT5G04590	Assimilatory sulfite reductase (ferredoxin)	1.3	17.0	10	CH
Q56YU0	AT3G24503	Aldehyde dehydrogenase family 2 member C4	1.3	10.6	4	C
Q9FLQ4	AT5G55070	2-Oxoglutarate dehydrogenase complex component E2-1	1.3	8.6	2	M
P52410-2	AT5G46290	Beta-ketoacyl-ACP synthase I	1.3	14.1	5	CH

*For each protein, UniProt ID, locus name, fold change, coverage and number of unique peptides is reported, together with their cellular localization (P, plasma membrane, C, cytoplasm; PO, peroxisome; CH, chloroplast; M, mitochondria; V, vacuole; CW, cell wall; CS, cytoskeleton; MM, membrane; EC, extra cellular; G, Golgi apparatus; EM, endo membrane; ER, endoplasmic reticulum).*

**Table 2:** List of proteins with decreased abundance in HS treated vs. untreated roots.

UniProt ID	Locus name	Description	Fold change	Coverage %	Unique peptides	Localization
Q96300	AT3G02520	14-3-3-Like protein GF14 nu	–1.3	42.3	3	N
P50700	AT4G11650	Osmotin-like protein OSM34	–1.3	48.4	9	EC
Q8LD03	AT5G16130	40S ribosomal protein S7-3	–1.3	41.1	6	C, MM, PM
P94014	AT1G09560	Germin-like protein subfamily 2 member 1	–1.3	21.0	4	
Q9SMW7	AT1G17880	BTF3b-like factor	–1.3	26.7	5	N
O49499	AT4G34050	Caffeoyl-CoA *O*-methyltransferase 1	–1.3	28.6	8	C
O04331	AT5G40770	Prohibitin-3, mitochondrial	–1.3	27.4	7	Nu, M, V, P,
Q9SIP1	AT2G31670	At2g31670	–1.3	23.2	6	PO, CH,
A8MR12	AT5G23540	26S proteasome non-ATPase regulatory subunit 14	–1.3	5.8	2	C
Q42342	AT5G53560	Cytochrome b5 isoform A	–1.4	44.0	7	V, ER, P, CH,
P17745	AT4G20360	Elongation factor Tu	–1.4	8.8	3	EC, Nu, CH,
Q9ZUG4	AT2G05830	Isoform 2 of Methylthioribose-1-phosphate isomerase	–1.4	11.4	3	EM
Q9C505	AT1G69410	Eukaryotic translation initiation factor 5A-3	–1.4	36.1	2	C
Q9FWR4	AT1G19570	Isoform 2 of Glutathione-*S*-transferase DHAR1, mitochondrial	–1.4	31.1	6	EC, M, V, PO, P, CH,
Q41931	AT1G62380	1-Aminocyclopropane-1-carboxylate oxidase 2	–1.4	27.8	6	CW
Q9ZRW8	AT1G78380	Glutathione-*S*-transferase U19	–1.4	39.7	11	P, CH,
Q9SRY5	AT1G02920	Glutathione-*S*-transferase F7	–1.4	55.5	6	V
O80858	AT2G30930	Expressed protein	–1.5	68.9	9	CH
Q96522	AT4G30170	Peroxidase 45	–1.5	71.1	17	EC, EM
B3H778	AT4G24830	Argininosuccinate synthase	–1.5	8.4	3	CH
P42760	AT1G02930	Glutathione-*S*-transferase F6	–1.5	52.9	6	CW, M, V,
Q43725	AT3G59760	Cysteine synthase, mitochondrial	–1.6	17.4	4	M, CH
Q42338	AT3G48140	AT3G48140 protein	–1.6	33.0	3	PO
P24704	AT1G08830	Superoxide dismutase [Cu-Zn] 1	–1.7	31.6	5	C
Q9FMA8	AT5G38940	Germin-like protein subfamily 1 member 11	–1.7	30.5	3	EC, CW
Q9SFF9	AT3G05950	Germin-like protein subfamily 1 member 7	–1.7	24.9	4	EC, EM
Q9FMA9	AT5G38930	Germin-like protein subfamily 1 member 10	–1.7	10.8	1	EC, CW,
Q9FIC6	AT5G39150	Germin-like protein subfamily 1 member 17	–2.2	15.4	2	EC, EM
P43296	AT4G39090	Cysteine proteinase RD19a	–2.2	14.4	4	V

*For each protein, UniProt ID, locus name, fold change, coverage and number of unique peptides is reported, together with their cellular localization (P, plasma membrane, C, cytoplasm; PO, peroxisome; CH, chloroplast; M, mitochondria; V, vacuole; CW, cell wall; CS, cytoskeleton; MM, membrane; EC, extra cellular; G, Golgi apparatus; EM, endo membrane; ER, endoplasmic reticulum).*

**FIGURE 2:**
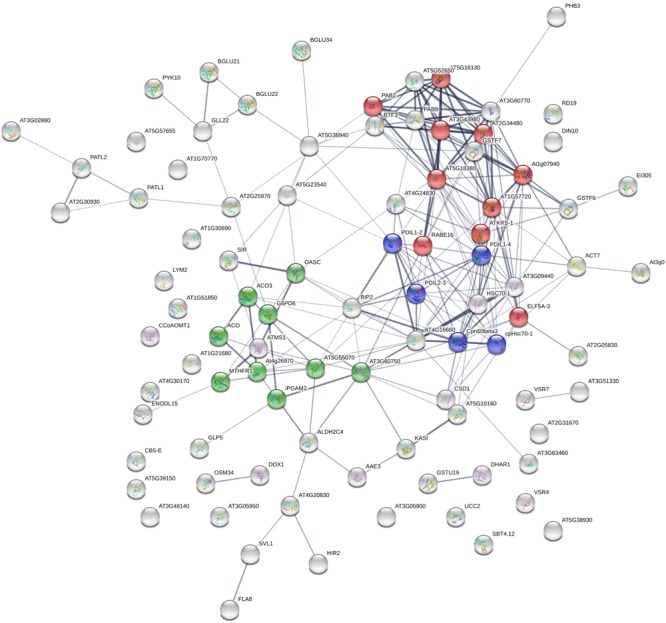
STRING analysis of 92 differentially expressed proteins following HS treatment, visualizing the presence of three major clusters. Colors indicate different biological function: red, protein synthesis; blue, protein folding and elongation; green, energy and metabolism (including secondary metabolism). Labels report protein Gene Names.

STRING analysis (Szklarczyk et al., 2017) reveals that most altered proteins are functionally connected (Figure 2) and three main clusters were identified, namely: protein synthesis, protein folding and elongation, energy and metabolism (including secondary metabolism).

**FIGURE 3:**
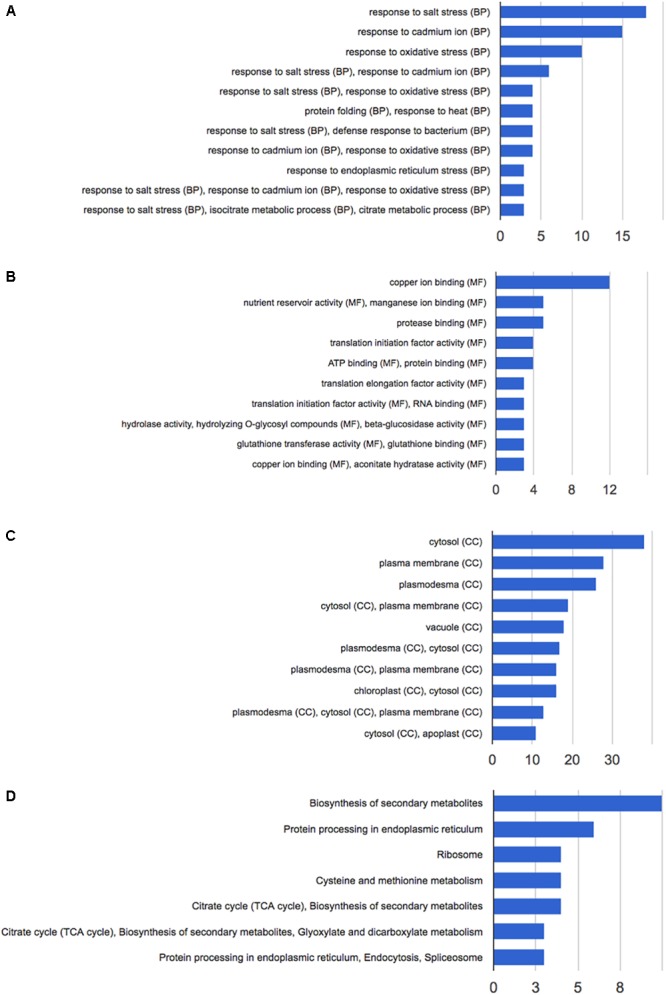
Functional annotation of the 92 differentially expressed proteins following HS treatment performed with GeneCodis 3. **(A)** Biological process; **(B)** molecular function; **(C)** cellular components; **(D)** KEGG pathways analysis.

The differentially expressed proteins were also subjected to Gene Ontology via GeneCodis3 platform analysis in order to identify major biological processes involved in the response to HS (Tabas-Madrid et al., 2012) (Figure 3).

Functional annotation analysis of the 92 altered proteins under high stringency conditions is reported in Figure 3A. Response to salt stress, response to cadmium ion and response to oxidative stress are the most enriched clusters. Other two major groups of altered proteins relate to protein folding and response to heat.

The main molecular functions highlighted by the analysis are copper and manganese ion binding, nutrient reservoir activity, and protease binding (Figure 3B). Most altered proteins belong to the cytosolic compartment, followed by plasma membrane, plasmodesma, vacuole, and apoplast (Figure 3C).

Pathway analysis and protein interaction network analysis were carried out to highlight the most regulated pathways and the interaction amongst the identified proteins. Most informative maps obtained in KEGG Pathway software are phenylpropanoid biosynthesis (KEGG:00940), protein processing in endoplasmic reticulum (KEGG:04141), ribosome (KEGG:03010), cysteine and methionine metabolism (KEGG:00270), glyoxylate and dicarboxylate metabolism (KEGG:00630) (Supplementary Figures S1–S5, respectively).

**FIGURE 4:**
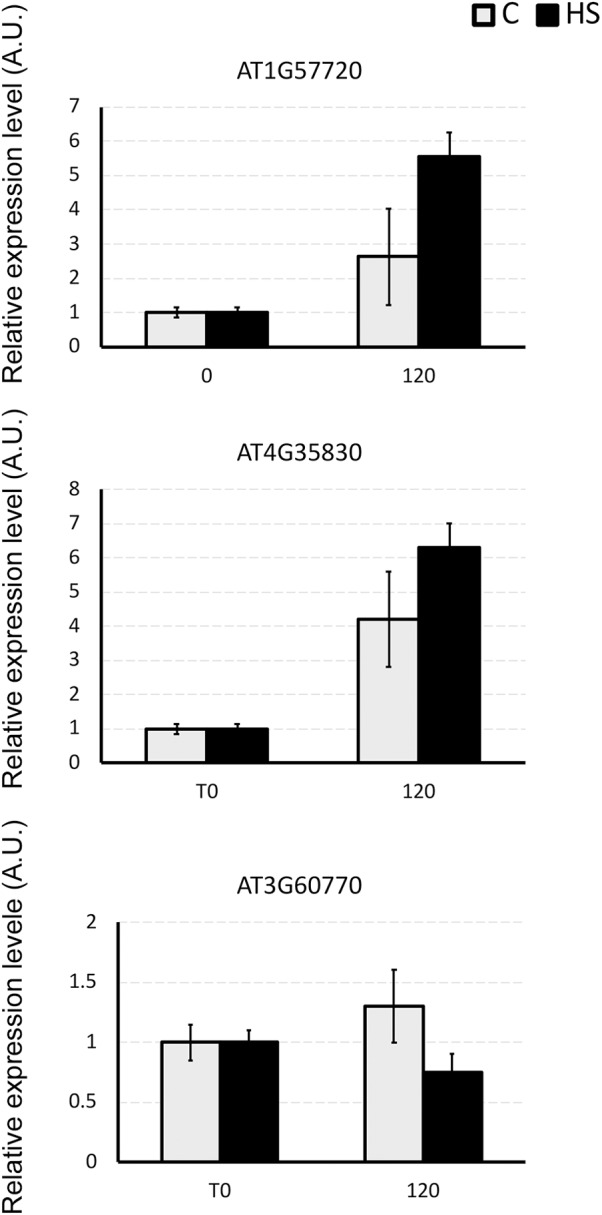
The relative expression level of AT1G57720, AT4G35830, and AT3G60770 genes in *Arabidopsis* roots under the whole nutrient (C) or after HS treatment (HS). The y-axis represents the relative transcript abundance ratios expressed in arbitrary units respect to the T_0_. Root samples were harvested 2 h after treatment, and mRNA abundance was determined by RT-qPCR. Error bars represented SD of three independent biological replicates.

Three genes encoding for differentially abundant proteins evidenced in this study, namely AT1G57720, probable elongation factor 1-gamma; AT4G35830, aconitate hydratase 1 and AT3G60770, 40S ribosomal protein S13-1, were selected to validate present results. Transcript levels of these genes were monitored by means of qPCR (Figure 4). The expression profiles of AT1G57720 and AT4G35830 fully confirmed the protein abundance patterns, evidencing an induction in transcript accumulation after 2 h of HS provision. The expression profile of AT3G60770 was demonstrated to be not directly affected by the presence of HS (2 h). In this case either the duration of the treatment was not sufficient to induce an up-regulation of the transcripts, or the protein may be controlled at the post-transcriptional level, confirming that changes in mRNA expression provide only limited insight into changes in protein expression.

## Discussion

**FIGURE 5:**
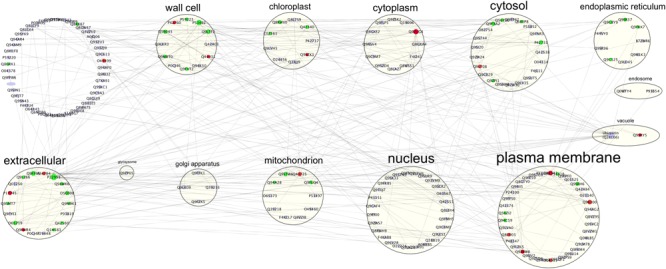
Interactome of 92 up- or down-regulated proteins detected in Arabidopsis roots treated with HS. The interactome created with IIS website included proteins with interactions between the input protein dataset and first-neighbor proteins. Proteins were visualized with Cytoscape 3.5.1 and they were distributed according to Cellular Components (GO) classification. Nodes marked in green were associated with significantly up-regulated proteins, red nodes denoted down-regulated proteins and grey nodes were first-neighbor proteins. Labels report protein UniProt ID.

Biostimulants and HSs ability to improve plant growth and development have been previously reported in different plant species (Nardi et al., 2009). In this case study increased protein content and higher root fresh weight (Figure 1) confirm the positive effect of HS treatment in plant physiology. To pinpoint the proteins involved in these responses iTRAQ proteomics has been adopted. However, the complex mechanisms occurring in the biological systems are not always fully grasped by means of the simple identification and quantification of proteins from a tissue (Carnielli et al., 2015). Protein–protein interactions and post-translational modifications (PTMs) are some of the numerous levels of complexity determining the life span, localization, and activity of a protein. This may play a pivotal role in regulating the transcriptional changes related to cellular and plant responses to stimuli (Walton et al., 2016). Thus, we explored the applicability of Integrated Interactome System (IIS, Carazzolle et al., 2014) and uploaded the UniProt protein IDs of differentially abundant Arabidopsis proteins in the IIS module. IIS networks provide information of proteins that might be interacting with the input list. This may contribute to a better understanding of the biological role of the HS-responsive proteins. Interactome network identified in our experimental setup evidenced that most of the proteins with different abundance are located in the extracellular; cell wall and plasma membrane (Figure 5) in the GO Cellular Components. This confirms that proteins associated with root cell plasma membrane can be a target for HS, and changes in their abundance may be seen as the primary reactions leading to the biological responses reported so far (Carletti et al., 2008).

**FIGURE 6:**
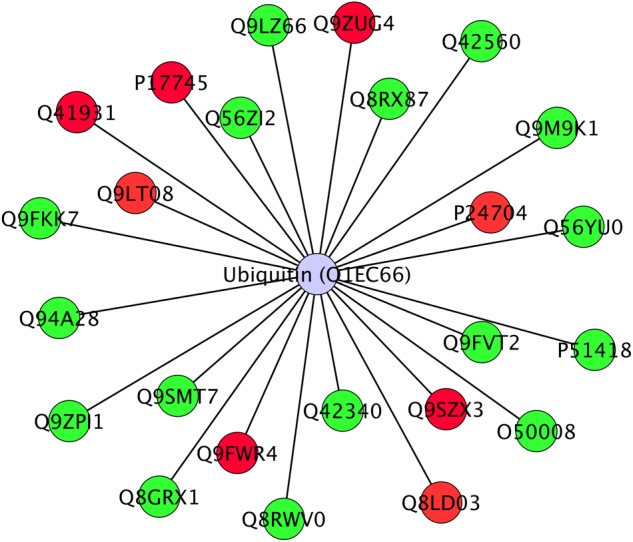
Interactome of 24 differentially abundant proteins detected in Arabidopsis roots treated with HS with known direct interaction with Ubiquitin in IIS database. Proteins were visualized with Cytoscape 3.4.0 and they were distributed with Ubiquitin (Ubq3) as central node. Nodes marked in green were associated with significantly up-regulated proteins, red nodes denoted down-regulated proteins. Labels report protein UniProt ID.

Twenty-four differentially abundant proteins (Figure 6), evidenced known protein–protein interactions with Ubiquitin (UBQ3). The small ubiquitin molecule attaches to lysine residues on target proteins, leading to the PMT named ubiquitination (Manzano et al., 2008; Igawa et al., 2009; Kim et al., 2013).

Ubiquitinated proteins have several different fates, the most common one being degradation by the 26S proteasome, but changes in their sub-cellular localization or activity are also potential fates. Ubiquitination is known to be involved in plant response to stress (Min et al., 2016) and in modulation of hormone signaling (Vierstra, 2009) as additional non-proteolytic function of ubiquitin modification (Stone, 2014). In our study, although not listed among identified proteins, ubiquitin presence in the interactome confirms its role in the responses that were triggered by HS and suggests that many of the regulatory processes might be controlled at post-translational levels.

In order to decipher the HS treatment-associated metabolic readjustments in roots, the altered proteins were discussed with reference to functional categories.

### Redox Homeostasis

We identified a protein related to redox homeostasis, protein disulfide isomerase 2 (AT1G77510), whose abundance increased in response to HS, while glutathione-*S*-transferase U19 (GSTU19) (AT1G78380), glutathione-*S*-transferase F7 (AT1G02920), Superoxide dismutase [Cu-Zn] 1, CSD1 (AT1G08830) were down-regulated by HS treatment. Reactive oxygen species such as hydrogen peroxide (H_2_O_2_), hydroxyl radical (OH^-^), superoxide ($O_{2}^{–}$) and singlet oxygen (^1^O_2_) have been considered as an unavoidable process of normal aerobic metabolism in plants (Dinakar et al., 2010). Different cellular molecules including proteins, DNA, RNA, and lipids may be destroyed by ROS (Shah et al., 2001), but plant cells have evolved enzymatic and non-enzymatic mechanisms against these deleterious effects of ROS (Zhang et al., 2009). Antioxidant enzymes such as catalase CAT (EC 1.11.1.6), CSD (EC 1.15.1.1), and peroxidase POD (EC 1.11.1.7) as well as other antioxidant small molecules (glutathione, ascorbic acid, and carotenoids) are used against oxidative stress by plants (Tewari et al., 2008).

Our results confirm the study of García et al. (2012) which showed that the antioxidant system of rice roots responds to HAs in a similar way as they do against stress. For example, the activity of CAT and POX were increased after 8 h of treatment with HA. Muscolo et al. (1993) also reported the morphogenetic influence of HS on leaf explant of *Nicotiana plumbaginifolia* due to stimulation of peroxidases and esterase. The study of Cordeiro et al. (2011) also presents the up-regulation of ROS and CAT in maize after the application of HA extracted from Oxisol. However, when applied in combination with environmental stresses, such as drought (de Vasconcelos et al., 2009) or salinity (Aydin et al., 2012), also opposite effects on these proteins have been observed.

Among the identified proteins, peroxidase isoform PER45 (AT4G30170) was found. This protein belongs to Class III peroxidases, comprising catalytically flexible enzymes with a great number of isoforms (a total of 73 in Arabidopsis), which have been found to regulate a wide range of physiological processes in plants such as cell wall metabolism, auxin catabolism, wound healing, generation of ROS. These enzymes use various electron donors such as phenolic compounds, auxin, secondary metabolites, or lignin precursors for reduction of H_2_O_2_ (Passardi et al., 2005).

Protein disulfide-isomerase 2-3 (AT2G32929) was up-regulated in our study; PDIs are enzymes of the oxidoreductase family and are involved in the formation, rearrangement, and reduction of disulfide bonds in proteins of eukaryotes (Cho et al., 2011). The proper folding of target proteins by PDI is necessary for the stability, trafficking, catalytic activity, and communication with other proteins; PDI has been seen to be involved in many different physiological processes and responses to various types of stresses (Lu and Christopher, 2008).

Another upregulated protein which may be involved in the protection against oxidative stress is Alpha-dioxygenase 1 (AT3G01420), catalyzing the primary oxygenation of fatty acids into oxylipins, a class of lipid-derived molecules including jasmonic acid, which are induced in response to salicylic acid and oxidative stress (Eckardt, 2008). This evidence also suggests the involvement of jasmonic acid in the signal transduction pathway leading to the hormone-like effects of HS.

Five Germin-like proteins (GLPs) have been identified among downregulated proteins (AT1G09560; AT5G38940; AT3G05950; AT5G38930; AT5G39150). Germin-like proteins (GLPs) compose a diverse family of plant glycoproteins belonging to the cupin superfamily. GLPs in cereals and other plant species may have oxalate oxidase (OXO) or superoxide dismutase (SOD) activity, producing H_2_O_2_ (Yasmin et al., 2015), however GLPs from different species were reported to be involved in biotic as well as abiotic stresses and also in the growth and development (Barman and Banerjee, 2015). For example, transgenic plants with increased levels of germin like protein subfamily 2 member 1 (AT1G09560) exhibited reduced primary root and enhanced lateral root growth suggesting a role as regulatory components of root architecture (Ham et al., 2012) in line with reported responses (Figure 1).

Many studies have demonstrated the regulatory role of ROS in many signaling pathways in plants both in redox homeostasis and under biotic stress (Mittler et al., 2011; Mittler, 2017). The discussed results on proteins involved in redox homeostasis, in particular SOD and glutathione-*S*-transferase, indicate an involvement in the response mechanisms induced by HS. These responses do not rule out the participation of the known hormonal signaling cascades in HS-induced responses, but suggests that ROS may play a pivotal role in regulating metabolic pathways related to plant growth, in synergy with auxin or NO stimuli. HS regulation of ROS metabolism in roots has been previously described (García et al., 2012, Garcia et al., 2016): present results are in agreement with the description of a situation of mild stress in HS-treated roots triggering the physiological responses leading to higher biomass and protein production (Olaetxea et al., 2018).

### Energy Metabolism/Respiration

A number of differentially abundant enzymes/proteins identified from Arabidopsis roots were found to be involved in plant energy metabolism.

Some of these enzymes are related to carbohydrate metabolism including glycolysis, TCA and pentose phosphate pathway and were more abundant such as aconitase 1 (AT4G35830), aconitase 2 (AT2G05710), aconitase 3 (AT4G26970), glucose-6-phosphate 1-dehydrogenase (AT5G40760). These results are resumed in KEGG Pathway (Supplementary Figure S3).

The glycolytic pathway is important in plants as it provides fuel for respiration and major carbon skeletons for the synthesis of various vital compounds such as nucleic acids, amino acids, fatty acids, isoprenoids, and other secondary metabolites (Plaxton, 1996). The role of glycolysis in opposing various stresses including drought, salt, cold, and anoxia has been widely reported in literature (Kosova et al., 2014). Thus, the known HS effect on plant stress relief (Aguiar et al., 2016) can be, at least partially, ascribed to their action on the glycolytic pathway.

One of the up-regulated enzymes in our study is glucose-6-phosphate dehydrogenase (G6PD) (AT5G40760), catalyzing the oxidation of glucose-6-phosphate to 6-phosphogluconate, the key step in the pentose phosphate pathway (PPP). PPP is the primary source of NADPH in various biosynthetic processes such as fatty acid metabolism, integration of nitrogen into amino acid and resistance against oxidative destruction. An intermediate in PPP, ribose-5-phosphate is used for phenylpropanoid production through shikimate pathway (Scharte et al., 2009). G6PD is highly regulated: besides transcriptional control, also redox regulation and cellular NADPH/NADP^+^ ratio have been shown to regulate the activity of diverse G6PD isoforms (Schurmann and Buchanan, 2008). In a previous study, the low molecular weight humic extracts were found to stimulate the Pi level and energetic metabolism, resulting specifically in higher glucose-6-phosphate and ATP level (Zancani et al., 2009).

In mitochondria, aconitase (ACO; EC 4.2.1.3) plays an important role in TCA cycle by catalyzing the isomerization reaction of citrate to isocitrate via *cis*-aconitate. The cytosolic isoform is involved in glyoxylate cycle (Moeder et al., 2007). We identified three isoforms of aconitase which were all upregulated: aconitase 1 (AT4G35830, cytoplasmic and mitochondrial), aconitase 2 (AT2G05710, mitochondrial) and aconitase 3 (AT4G26970, mitochondrial). Adjacent spots of aconitase were also reported as differentially expressed by Carletti et al. (2008) in 2D gels of HS-treated maize roots.

Taken together, the enhanced abundance of the above-mentioned enzymes involved in glycolysis, pentose phosphate pathway, and TCA cycle due to HS may result in an increase in the production of NAD(P)H, ATP and carbon skeletons needed for various vital cellular processes such as biosynthesis of macromolecules (proteins, nucleic acids, amino acids, fatty acids, secondary metabolites), which, in turn may explain the known effect of HS on plant growth. In analogous experimental conditions, using the same plant species and same HS treatment, metabolomics results evidenced a significative decrease (around -50%) in carbohydrates abundances in roots (Conselvan et al., 2018). Lower contents of fructose, glucose and sucrose represent a metabolic confirmation of the proteomic results in our study, and substantiate the hypothesis of enhanced glycolysis in HS-treated plants.

The stimulation of energy metabolism related enzymes after HS treatment has been reported previously by many researchers: up-regulation of various metabolic processes and signaling pathways associated with plant development (Trevisan et al., 2010; Pizzeghello et al., 2013), stimulation of glycolysis and TCA cycle related enzymes in maize (Nardi et al., 2007). Quaggiotti et al. (2004) also found that HS can stimulate carbon and nitrogen metabolism by overexpression of various enzymes of glycolysis and TCA cycle.

### Cell Wall Metabolism

Cell wall is contained in the outermost extracellular matrix in plant cells and its regulation is important for proper size and shape, mechanical resistance, interaction with environment, defense against pathogens, development and growth (Reiter, 2002). Cell wall is the first compartment getting in contact with the exogenous agents, thus unsurprisingly its proteome is altered by the HS-treatment. In our study, many of identified enzymes related to cell wall metabolism were up-regulated (Figure 2), as for example the enzyme glycerophosphodiester phosphodiesterase (GDPD) (AT5G55480) (EC 3.1.4.46). This enzyme plays a vital role in many physiological processes in living organisms, by converting glycerophosphodiester to glycerol-3-phosphate and alcohols during glycerol metabolism (Cheng et al., 2011). GDPD and its homologs have been found to be involved in cell wall organization and in root hair morphogenesis in Arabidopsis (Hayashi et al., 2008).

These evidences point to readjustments in cell wall composition which are likely required in remodeling and redefining the root organ size, architecture and root hair morphogenesis when stimulated by HS, as found in Trevisan et al. (2010).

### Protein Synthesis, Folding, and Degradation

The involvement of HS in stimulation of protein synthesis in plants has been previously observed in many studies: in Arabidopsis roots (Trevisan et al., 2011), maize roots (Carletti et al., 2008), and guava leaves (Dantas et al., 2007). Canellas et al. (2002) also observed that the HA and Indole Acetic Acid (IAA) groups may be able to access receptors and resulted in activation of protein synthesis in maize roots.

Our identified proteins comprise 40 and 60S ribosomal proteins (RPs) mostly up regulated (AT2G34480, AT5G18380, AT4G33865, AT3G60770, AT5G52650 up-regulated; AT5G16130 down-regulated). Ribosomes are the basic and essential components of every cell and catalyze numerous transpeptidal esterase reactions during protein synthesis. Ribosomal proteins (RPs) are not only vital for protein synthesis but also play a central role in cell division, growth, and metabolism. The role of RPs as regulatory components in addition to their housekeeping function in developmental processes has been indicated by various mutational studies (Byrne, 2009).

Ribosomal proteins are regulated by various growth regulators: for example, the application of BAP (cytokinin) and IAA (auxin) increased the transcription of RPS15aF while abscisic acid (ABA) treatment decreased it. Abiotic stresses like temperature and mechanical stress increased the transcript of RPS15aA, RPS15aD, and RPS15aF (Hulm et al., 2005).

This could suggest an overall increase in ribosome production in HS treated root, which is consistent with enhanced protein synthesis. Protein contents, both in root and leaves (Figures 1D,E) corroborate these proteomic results. Moreover, enhanced protein synthesis is also confirmed by the lower concentration of free amino acids in HS-treated plant roots grown in analogous conditions. All identified free amino acids were less abundant (ranging from -30 to -60%) compared to control plants (Conselvan et al., 2018). Decrease in free amino acid content, paired with higher abundance of ribosomal proteins and higher total protein content account for plant root responses leading to higher biomass production (Figure 1B).

The enzyme lysyl-tRNA synthetase (AT3G11710), which is up-regulated in our experiment, has a key role in conversion of genetic information from mRNA to protein by catalyzing the formation of lysyl-tRNA (Freist and Gauss, 1995). The enzyme is also found to be linked with many other secondary functions such as activation of gene expression (Lee et al., 2004), and by serving as a cytokine (Park et al., 2005).

Our data report an increase of two isoforms of poly(A)-binding protein (PABP2, AT1G49760; AT4G34110). After attaching with 3′ end of mRNA, PABP interacts with eukaryotic translation initiation factor 4F complex enhancing the translation process inside the cell (Sachs and Varani, 2000). The role of PABP is also found to be correlated with nuclear export of mRNAs (Brune et al., 2005) and stability (Behm-Ansmant et al., 2007). In *A. thaliana*, a total of eight different isoforms of PABP have been characterized (Belostotsky, 2003).

Various heat shock protein cognates (AT4G24280, AT3G09440) were also over produced in response to HS. The 70-kD heat shock proteins (Hsp70s) are found in all cellular compartments of almost all organisms and have been found to be crucial for protein folding, protein translocation, and stress responses (Latijnhouwers et al., 2010). One of the up-regulated Hsp70s have been described in Arabidopsis as stromal isoforms cpHsc70-1 (At4g24280). In one knockout study a mutation in cpHsc70-1 resulted in abnormal leaves, impaired root development and retardation in growth (Su and Li, 2008).

Plant proteasomes (26S proteasomes) contain two subparticles: the core particle (CP) or 20S proteasome where proteins are degraded, and the regulatory particle (RP) or 19S proteasome. 26S proteasomes are in charge of the ATP-dependent degradation of ubiquitin tagged proteins (Sadanandom et al., 2012) including normal, mutated, misfolded, and damaged proteins (Kurepa and Smalle, 2008; Tolin et al., 2013). The ubiquitin-proteasome system plays a role in nearly all aspects of cell homeostasis including plant development, response to plant hormones (Sullivan et al., 2003) and signaling in response to abiotic and biotic stimuli (Smalle and Vierstra, 2004). Moreover, the 20S particle can also degrade proteins in a ubiquitin-independent manner, mainly for oxidized proteins (Foyer and Allen, 2003). In this study HS treatment resulted in differential abundance of a proteasome subunit such as 26S proteasome non-ATPase regulatory subunit 14 (AT5G23540). The involvement of the proteasome complex may be seen as a component of cell metabolic remodeling in the response to HS treatment, coherently with the view that the proteolytic capacity of a cell is the result of a careful balancing act that reflects environmental conditions and developmental stage (Kurepa and Smalle, 2008).

26S proteasome is ubiquitin and ATP dependent, and is involved in protein degradation in the nucleus and the cytosol (Voges et al., 1999; Pickart and Cohen, 2004). Various catalytic activities such as trypsin-like, chymotrypsin-like peptidylglutamyl-peptide hydrolase are performed by 20S core particle of 26S proteasome (Groll et al., 1997; Voges et al., 1999).

### Cell Trafficking and Division

Our proteomic data identified proteins related to cell vesicle trafficking and growth such as Actin-7 (AT5G09810), Patellin-1 (AT1G72150), Patellin-2 (AT1G22530), Patellin-4 (AT1G30690), all of which were up-regulated. One other protein involved in actin metabolism was also found to be upregulated, namely GDPDL4 (AT5G55480), reported to be involved in actin nucleation (Ma et al., 2012). The involvement of actin in plant responses to HS has been already highlighted in previous works (Carletti et al., 2008) as well as transport processes and vesicles trafficking related genes (Trevisan et al., 2011).

The role of actin in cellular processes is diverse and ranges from cell division and morphogenesis to cell motility (Pollard and Cooper, 2009). Actin is also found to be important for tip growth (polarized cell extension) in plants (Menand et al., 2007), whose implications are of particular relevance in this study.

Patellin is a phosphoinositide-binding protein that plays a role in membrane trafficking during the expansion and maturation stages of cytokinesis, in particular cell-plate formation (McMichael and Bednarek, 2013).

### Response to Inorganic Substances

In this study, we identified a number of proteins as responses to inorganic substances that were affected by HS treatment. Many of these are also involved in redox homeostasis, such as glutathione-*S*-transferase 1 (AT1G02930), Peroxidase 45 (AT4G30170), and [Cu-Zn] Superoxide dismutase (AT1G08830) (details in redox homeostasis paragraph).

The group also contains some of the enzymes of glycolysis and TCA cycle as aconitate hydratase 2 (AT2G05710), Aconitate hydratase 3 (AT4G26970). Similarly, some other identified proteins of diverse function were over expressed as heat shock cognate 70 kDa protein 1 (AT5G02500), heat shock cognate 70 kDa protein 3 (AT3G09440), protein disulfide-isomerase 2 (AT1G77510).

### Heat Response

Humic substance caused a significant increase in abundance of two heat responsive proteins in this study: heat shock 70 kDa protein 6 (AT4G24280); heat shock 70 kDa protein 3 (AT3G09440).

The 70-kD heat shock proteins (Hsp70s) are molecular chaperones involved in a variety of cellular processes including protein folding, protein transport across membranes, modulation of protein activity, regulation of protein degradation, and prevention of irreversible protein aggregation. Plant Hsp70s are encoded by a multiple-gene family (Su and Li, 2008). It is well-known that Hsps are ubiquitous proteins found in plant and animal cells, which were initially described to be involved in heat shock, but they are known to be induced by a wide variety of stresses, including cold, drought, salt, UV-light, wound, and biotic stresses (Wen et al., 2017).

### Integration With Previous Results

In this work we have provided a thorough proteomic analysis in Arabidopsis roots treated with HS obtained at our laboratory, following a standardized procedure for extraction, purification, and characterization providing a product with homogeneous and stable properties over time.

Identical experimental conditions in terms of HS quality, concentration, exposure time, growth chamber settings (temperature, humidity, daylength) were previously used to treat Arabidopsis plants also in another study, aimed at studying the effects of HS on gene expression by a transcriptomic approach based on the detection of cDNA-AFLP markers (Trevisan et al., 2011).

**FIGURE 7:**
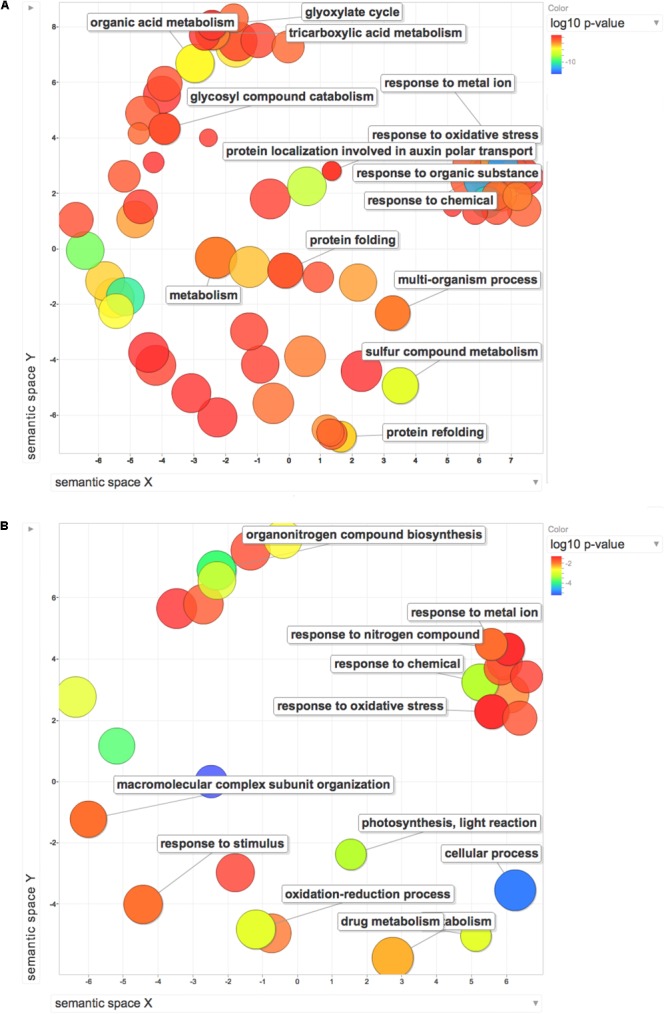
REViGO Scatterplot of the Enriched GO Terms representatives for the differentially regulated proteins **(A)** and DEGs **(B)** isolated by Trevisan et al. (2010). Bubble color indicates the *p*-value (legend in upper right-hand corner), the two ends of the colors are red and blue, depicting lower- and higher *p*-values respectively. Size indicates the relative frequency of the GO term in the underlying reference database. Bubbles of more general terms are larger.

Gene ontology enrichment analysis was used to compare the effect exerted by HS on proteome and transcriptome regulation. The terms along with their *p*-values were further summarized independently by the REViGO^4^ reduction analysis tool that condenses the GO description by removing redundant terms. Results of these further reductions are visualized in Figure 6. An encouragingly simple relationship between changes of transcripts and proteins and changes in downstream biological functions could be inferred by the graphs. Analysis on transcripts retrieved 32 GO terms, whilst the enrichment analysis on dysregulated proteins identified 116 terms. Despite the differences in the amount of enriched terms, the comprehensive proteome (Figure 7A) and transcriptome (Figure 7B) datasets demonstrated that several of the enriched GO terms (carbohydrate metabolic process, organic substance biosynthetic process, response to chemical, response to oxidative stress, response to abiotic stimulus, translation) are included in both the enriched groups, suggesting some correlation between transcript and protein levels.

## Conclusion

With the present work, an overview of metabolic pathways influenced by HS activity is presented, only in part previously observed. Our results, also in accordance with previously published metabolomic data, point to the activation of enzymes involved in glycolysis, pentose phosphate pathway and TCA cycle to support the production of NAD(P)H, ATP, and carbon skeletons needed for various vital cellular processes. Stimulation of energy metabolism may explain the known beneficial effects of HS on plant growth. Up-regulation of ribosomal proteins and actin are representative of a co-ordinately enhanced protein synthesis, folding, trafficking and transport across membranes which is required to sustain growth. Our findings also point to readjustments in cell wall composition which are required in root remodeling and root hair morphogenesis. The regulation of ROS-related enzymes indicates that these compounds play a pivotal role in response to HS stimulus, possibly acting as a regulatory mechanism to coordinate the other responses leading to growth enhancement.

The results discussed in this study should represent a new framework in the development of a new model mechanism, considering ROS as a chemical species of great importance in the action of HS on plants.

## Author Contributions

SR and PC wrote the manuscript with contribution from all authors. GC and SR analyzed the data. MP performed the protein extraction and purification. GA performed all proteomics experiments and statistical analysis. AM and TY contributed in the interpretation of the results and revised the manuscript. ST and SQ performed the transcriptome analyses. PC conceived the original idea and supervised the project. All authors discussed the results and contributed to the final manuscript.

## Conflict of Interest Statement

The authors declare that the research was conducted in the absence of any commercial or financial relationships that could be construed as a potential conflict of interest. The handling Editor is currently co-organizing a Research Topic with one of the authors, AM, and confirms the absence of any other collaboration.

## Footnotes

1 http://bioinfo.ut.ee/primer3-0.4.0/

2 http://www.genome.jp/kegg/tool/map_pathway2.html

3 http://bioinfo03.ibi.unicamp.br/lnbio/IIS2/index.php

4 http://revigo.irb.hr/

## Supplementary Material

FIGURE S1 Regulatory changes on the pathways of cysteine and methionine metabolism, with highlighted up- regulated proteins (green) and down-regulated proteins (red). Labels report EC numbers. The image was obtained by KEGG platform (https://www.kegg.jp; see reference in the text).

FIGURE S2 Regulatory changes on the pathways of glyoxylate and dicarboxylate metabolism, with highlighted up- regulated proteins (green). Labels report EC numbers. The image was obtained by KEGG platform (https://www.kegg.jp; see reference in the text).

FIGURE S3 Regulatory changes on the pathways on phenylpropanoid biosynthesis, with highlighted up- regulated proteins (green) and down-regulated proteins (red). Labels report EC numbers. The image was obtained by KEGG platform (https://www.kegg.jp; see reference in the text).

FIGURE S4 Regulatory changes on ribosome proteins, with highlighted up- regulated proteins (green) and down-regulated proteins (red). Labels report EC numbers. The image was obtained by KEGG platform (https://www.kegg.jp; see reference in the text).

FIGURE S5 Regulatory changes on protein processing in endoplasmic reticulum, with highlighted up- regulated proteins (green) and down-regulated proteins (red). Labels report EC numbers. The image was obtained by KEGG platform (https://www.kegg.jp; see reference in the text).

TABLEE S1 **List of differentially abundant proteins**. Table lists all proteins with a significantly different abundance in HS vs CTRL samples. Uniprot accession number, gene name, protein description, quantification values relative to the 3 replicates, fold change, and p value are reported.

TABLEE S2 **List of all identified peptides**. Table lists all peptides identified by the LC-MS/MS analysis together with all relevant parameters required to assess peptide identification and quantification confidence.

TABLEE S3 **List of all identified proteins**. Table lists all proteins identified with high confidence by the LC-MS/MS analysis, together with all relevant parameters required to assess protein identification and quantification confidence.
