# Supplementary figures and images for: Protein Profiling of Arabidopsis Roots Treated With Humic Substances: Insights Into the Metabolic and Interactome Networks
Source: Front Plant Sci. 2018 Dec 12;9:1812. doi: 10.3389/fpls.2018.01812 (PMC6299182; doi:10.3389/fpls.2018.01812)

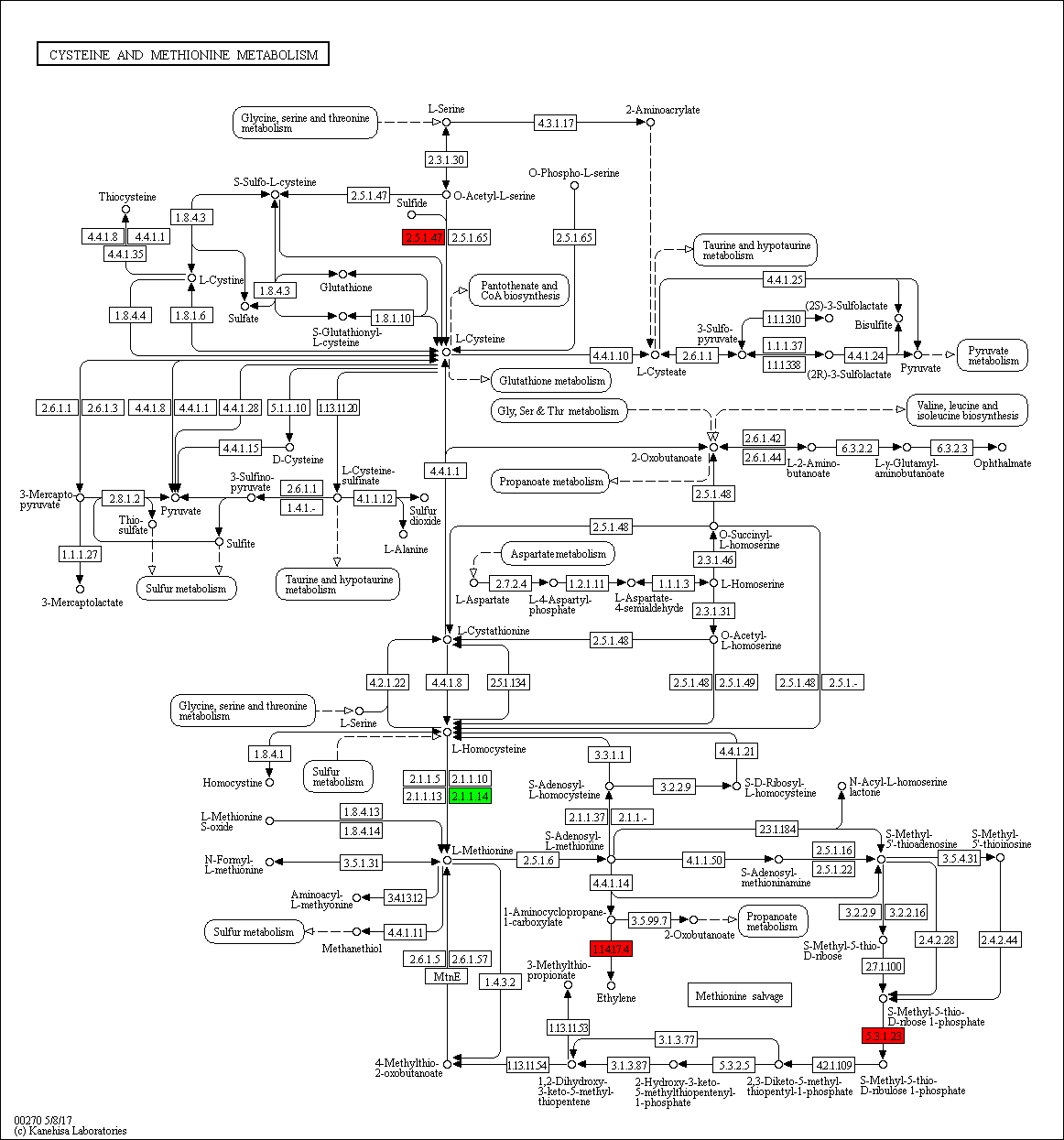

Supplement: FIGURE S1 — Regulatory changes on the pathways of cysteine and methionine metabolism, with highlighted up- regulated proteins (green) and down-regulated proteins (red). Labels report EC numbers. The image was obtained by KEGG platform (https://www.kegg.jp; see reference in the text). [file Image_1.PNG]

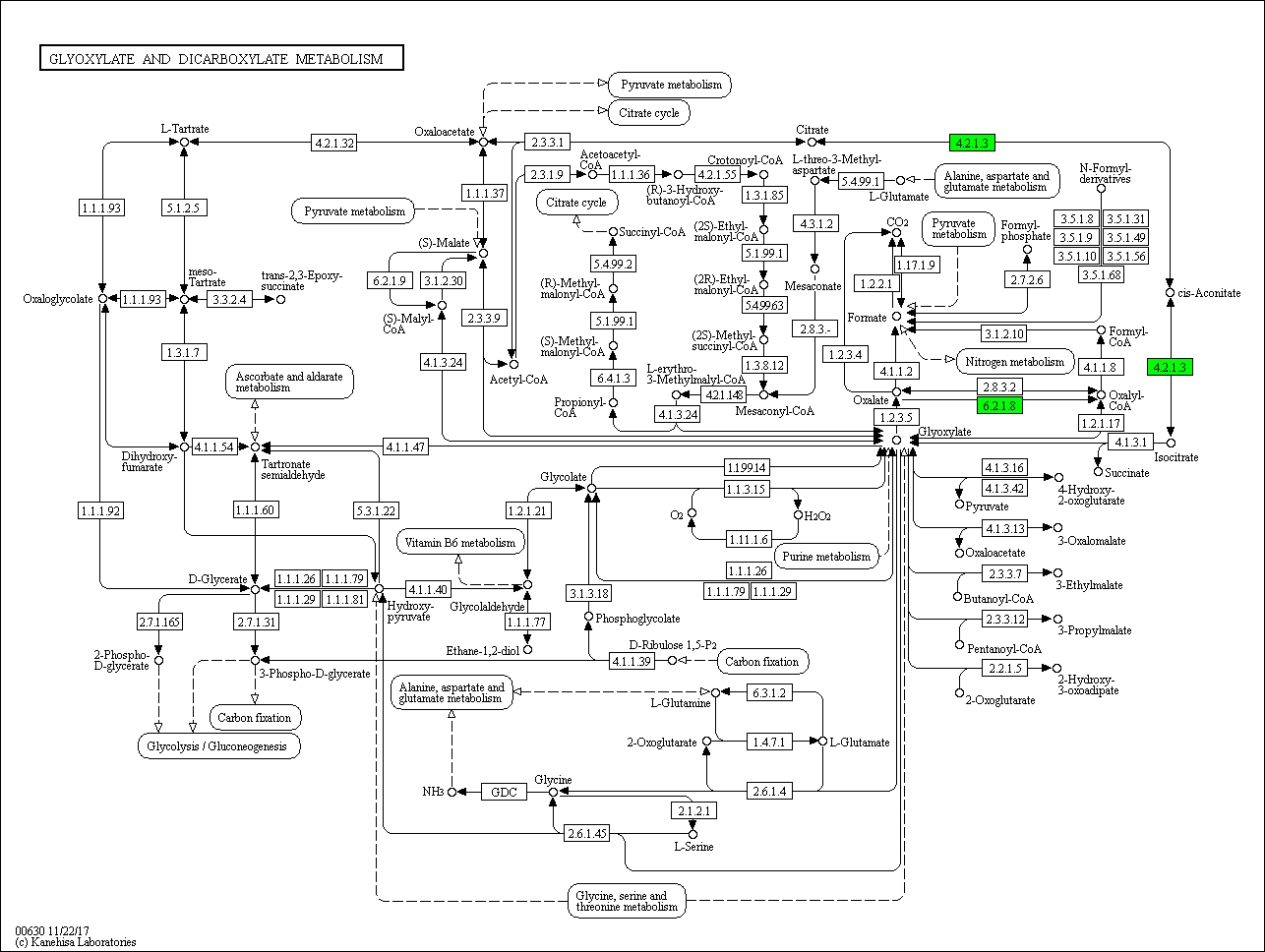

Supplement: FIGURE S2 — Regulatory changes on the pathways of glyoxylate and dicarboxylate metabolism, with highlighted up- regulated proteins (green). Labels report EC numbers. The image was obtained by KEGG platform (https://www.kegg.jp; see reference in the text). [file Image_2.PNG]

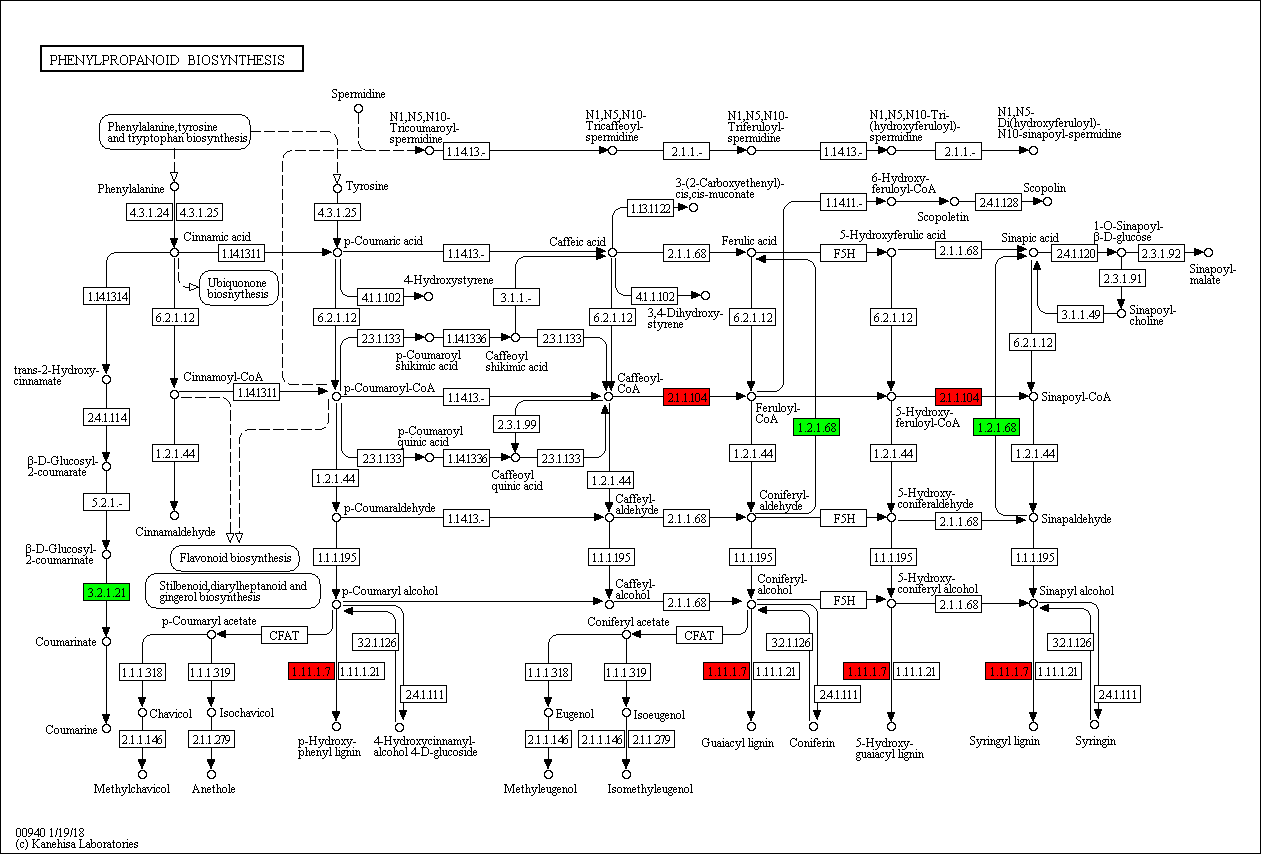

Supplement: FIGURE S3 — Regulatory changes on the pathways on phenylpropanoid biosynthesis, with highlighted up- regulated proteins (green) and down-regulated proteins (red). Labels report EC numbers. The image was obtained by KEGG platform (https://www.kegg.jp; see reference in the text). [file Image_3.PNG]

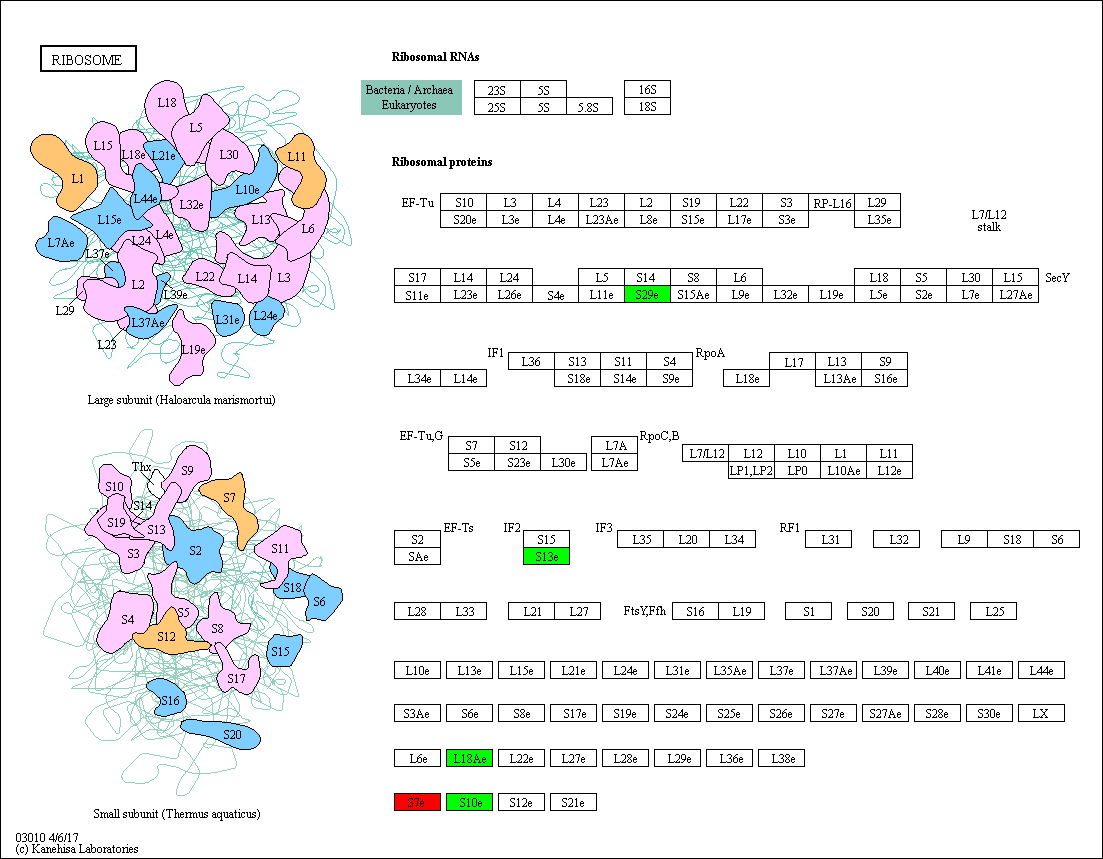

Supplement: FIGURE S4 — Regulatory changes on ribosome proteins, with highlighted up- regulated proteins (green) and down-regulated proteins (red). Labels report EC numbers. The image was obtained by KEGG platform (https://www.kegg.jp; see reference in the text). [file Image_4.PNG]

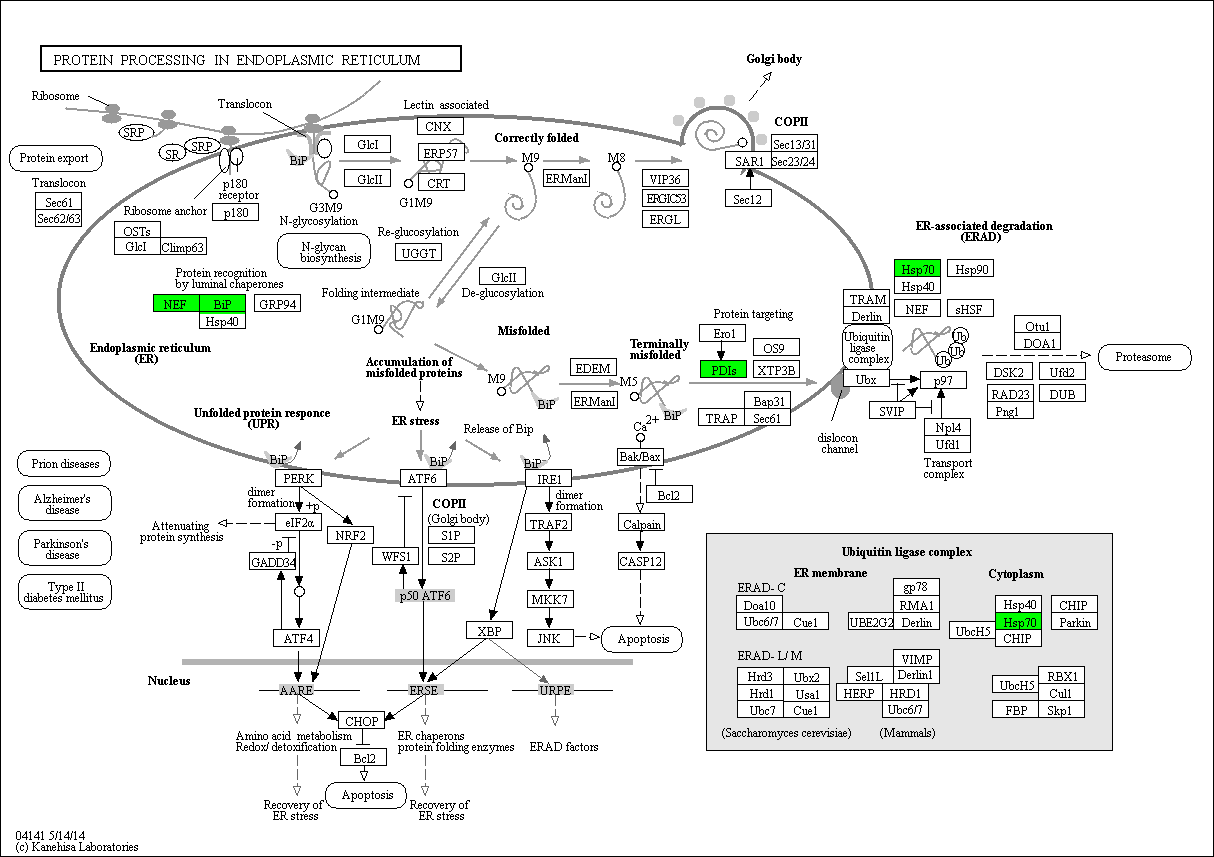

Supplement: FIGURE S5 — Regulatory changes on protein processing in endoplasmic reticulum, with highlighted up- regulated proteins (green) and down-regulated proteins (red). Labels report EC numbers. The image was obtained by KEGG platform (https://www.kegg.jp; see reference in the text). [file Image_5.PNG]
